# High performance platinum contacts on high-flux CdZnTe detectors

**DOI:** 10.1038/s41598-023-45331-9

**Published:** 2023-10-20

**Authors:** Manuele Bettelli, Silvia Zanettini, Leonardo Abbene, Francesca Casoli, Lucia Nasi, Giovanna Trevisi, Fabio Principato, Antonino Buttacavoli, Andrea Zappettini

**Affiliations:** 1 IMEM-CNR, 43124 Parma, Italy; 2 due2lab S.R.L., 42019 Scandiano, RE Italy; 3 Department of Physics and Chemistry (DiFC) - Emilio Segrè, University of Palermo, 90128 Palermo, Italy

**Keywords:** Materials for devices, Physics

## Abstract

The need for direct X-ray detection under high photon flux with moderate or high energies (30–100 keV range) has strongly increased with the rise of the 4th Generation Synchrotron Light Sources, characterised by extremely brilliant beamlines, and of other applications such as spectral computed tomography in medicine and non-destructive tests for industry. The novel Cadmium Zinc Telluride (CZT) developed by Redlen Technologies can be considered the reference material for high-flux applications (HF-CZT). The enhanced charge transport properties of the holes allow the mitigation of the effects of radiation induced polarization phenomena, typically observed in standard CZT materials (LF-CZT) under high photon flux. However, standard LF-CZT electrical contacts led to inacceptable high dark leakage currents on HF-CZT devices. In this work, a detailed study on the characteristics of new optimized sputtered platinum electrical contacts on HF-CZT detectors is reported. The results from electrical and spectroscopic investigations, showed the best performances on HF-CZT detectors with platinum anode, coupled with both platinum or gold cathode. The morphology, structure, and composition of Pt/CZT contact have been analysed by means of Transmission Electron Microscopy (TEM) on microscopic lamellas obtained by Focused Ion Beam (FIB), highlighting the presence of CdTeO_3_ oxide at the metal semiconductor interface.

## Introduction

Despite several decades of progress in developing CdZnTe-based radiation detectors, both crystal growth and contact deposition can still be considered hot topics in this field of research. Recently, Redlen Technologies (Canada) proposed a new grade of CdZnTe material able to withstand high radiation fluxes to satisfy the ever-growing needs of application fields like medical and security imaging^1^. The high-flux CdZnTe (HF-CZT) can operate at continuous X-ray fluxes > 10^8^ photons/mm^2^/s^2^ and at far higher levels of instantaneous flux tested with extreme intensities delivered by a free electron laser (FEL)^3^. HF-CZT is also characterized by excellent spatial uniformity^4^, outstanding linear response and time stability at fluxes up to 10^10^ photons/mm^2^/s^5^ as was recently tested. It has been well demonstrated by now that such astonishing performances at intense radiation fluxes are related to a hole’s lifetime increase of an order of magnitude (${\tau }_{h}\sim 2.5\, \upmu s$)^6–8^ with respect to standard grade CZT (LF-CZT) and a hole’s mobility-lifetime product ${{\mu }_{h}\tau }_{h}$ exceeding 10^–4^ cm^2^/V^9^.

The development and improvement of new contact deposition techniques is stimulated by the availability of new promising materials. One of the main challenges posed by CdZnTe is the fact that the same contact deposition procedure may lead to strongly different results on CZT crystals with slightly different physico-chemical properties, trap characteristics and Fermi-level position^10^. Selecting the appropriate electrode material is essential for minimizing the leakage currents. Despite several studies have been carried out reporting electrical characterization of contacts on standard LF-CZT detectors^11–16^, only few studies were performed on HF-CZT^17^.

The first attempts in realising blocking contacts on this material by means of the technique developed at IMEM-CNR, i.e., gold electroless deposition technique from alcoholic solutions^13^, were unsuccessful. This deposition technique allowed to obtain performing and robust contacts on both boron-encapsulated vertical-Bridgman grown CZT and Redlen LF-CZT, as shown in previous publications^18–20^, but producing sensors with extremely high leakage currents even at low bias voltage when used on HF-CZT. The platinum electroless contacts developed by IMEM-CNR and reported in Bettelli et al.^16^ were also tested. However, similarly to the gold electroless contacts, these contacts induced excessively high leakage currents, making them unsuitable for radiation detector applications.

This limitation stimulated the development of new optimized contacts for HF-CZT detectors. In this context, we developed new platinum sputtered contacts, obtaining HF-CZT detectors with low leakage currents and interesting spectroscopic performance.

## Methods

### Sample preparation

Several detectors were fabricated on HF-CZT, with different electrode configurations. HF-CZT crystals, provided by Redlen Technologies, are oriented along the <111> crystallographic direction and have two polar faces, A-face or Cd-face (cadmium-rich face) and B-face or Te-face (tellurium-rich face). Usually Cd-face is more p-type and is conventionally chosen as anode, while Te-face is more n-type and typically chosen as cathode^21^. As discussed in the introduction, the transport properties of HF-CZT differ from those of standard spectroscopic CZT (LF-CZT), with mobility-lifetime products of 10^–3^ and 10^–4^ cm^2^/V for electrons and holes, respectively^6^.

Detectors equipped with platinum sputtered contacts and gold electroless contacts were realised. Platinum contacts were deposited at IMEM-CNR using the sputtering technique (base vacuum: 3 × 10^–8^ mbar; Ar pressure: 8 × 10^–3^ mbar, target voltage: 1000 V). The resulting Pt layer appears homogeneous, shiny, and off-white. The layer thickness is about 100 nm, the surface roughness is 5.0 nm and 4.0 nm for R_q_ and R_a_ respectively as measured with AFM (see Fig. SI_1 in Supplementary Info). Gold contacts were instead deposited following the electroless procedure developed by Benassi et al*.*^13^ that ensures high mechanical stability.

**Figure 1:**
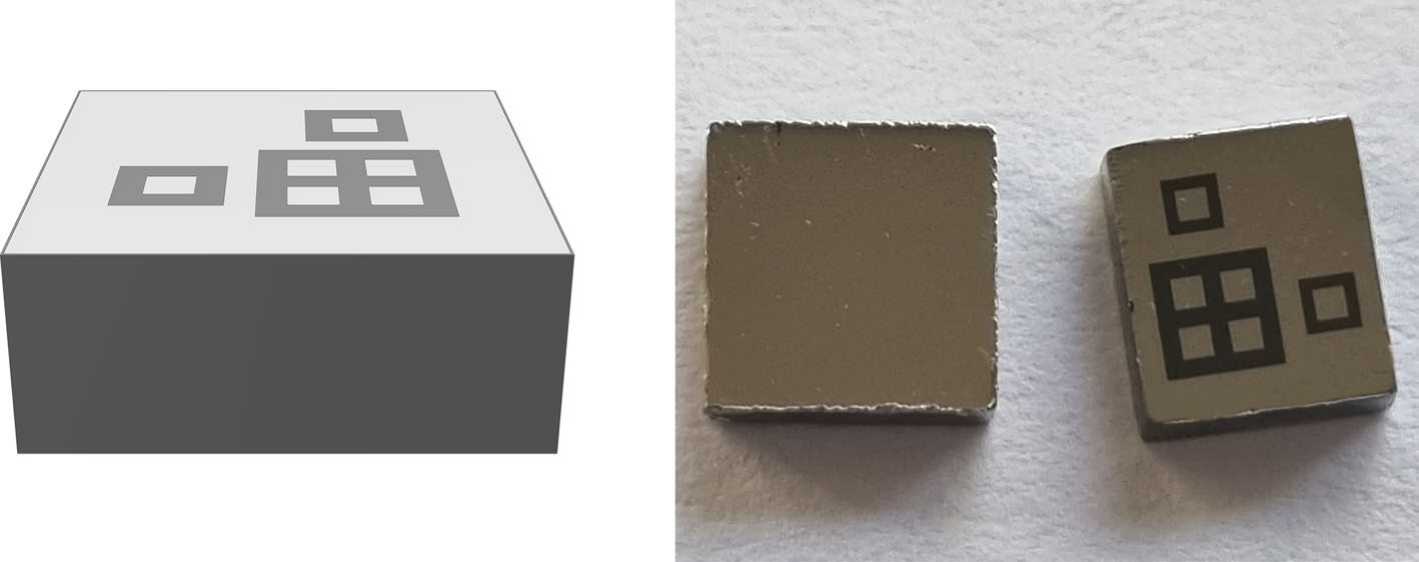
Ramm3D model of Pt anode (left); photograph of the Pt full-area cathode and the pixellated Pt anode (right).

Four detectors were fabricated starting from a single chip of HF-CZT cut into four 5 × 5 × 1.5 mm^3^ crystals oriented along the <111> direction. Each sample was lapped and polished before depositing the contacts. The detectors had a full-area cathode and a customised pixelated anode as shown in Fig. 1. Pixel size is 500 µm, gaps between pixels and guardpad are 200 µm large. Pixels area is 0.25 mm^2^. The pattern is composed of two single isolated pixels (surrounded by guardpad) and a 2 × 2 pixel matrix. The four detectors had the same geometry (Fig. 1) but different combinations of metal contacts at the anode (Cd-face) and cathode (Te-face). We carried out a comparative study to evaluate their characteristics.

All possible electrode configurations were tested and, subsequently, the best detectors were investigated extensively to characterise their behaviour. Samples are named reporting their cathode/CZT/anode structure in the following way:

1. Pt/CZT/Pt detector, where both contacts were made of platinum, is called PP.
2. Au/CZT/Au detector, where both contacts were gold, is called AA.
3. Au/CZT/Pt detector, where cathode was gold, while anode was platinum, is called AP.
4. Pt/CZT/Au detector, where cathode was platinum while anode was gold, is called PA.

### Current–voltage measurements and modelling

Preliminary electrical characterization was performed at IMEM-CNR Parma (Italy), in order to first check leakage currents of the detectors. Current–voltage (I–V) measurements were carried out in a probe station equipped with a metallic plate and two probe tips mounted on micromanipulators and connected to a Keithley 2410 sourcemeter and a Keithley 6548 picoammeter. The system is located inside a Faraday cage that allows to ensure dark measurement conditions and to reach very low noise levels.

To investigate the electrical properties of the detectors at different temperatures, a second set of electrical measurements was performed at the Department of Physics and Chemistry (DiFC) of University of Palermo by using a temperature-controlled system. The I–V curves were measured with the CAEN NDT1471 power supply connected to the cathode and the Keithley 2635B, configured as an electrometer, connected to the pixel anode. The guard electrode was forced to the ground potential. I–V measurements were performed in both reverse (i.e., by applying a negative voltage to the full-area electrode) and forward biasing (i.e., by applying a positive voltage to the full-area electrode). All measurements were performed with the detectors enclosed in a shielded box under a nitrogen atmosphere with a temperature control system.

To measure the main characteristic parameters of the electrical contacts (barrier height, barrier lowering, interfacial layer properties), we analysed the measured I–V curves through the application of a new procedure reported in Ref.^17^. This method allows to extract contact characteristic parameters in metal/semiconductor structure, such as CZT detectors, where the electrical currents follow the interfacial layer–thermionic-diffusion (ITD) model theory^22,23^. According to this model, in the voltage bias range where the transport mechanism is dominated by the metal/semiconductor junction, the current density $J$ can be written as:

1 $$J=\frac{{A}^{*}\theta }{1+\frac{\theta {V}_{R}}{{V}_{D}}}{e}^{-\frac{{\phi }_{{B}_{0}}}{{V}_{TH}}}{e}^{\frac{{C}_{2}V}{{V}_{TH}}},$$

where $V$ is the reverse bias voltage, ${V}_{TH}=kT/q$, ${\varphi }_{B0}$ is the barrier height under thermal equilibrium conditions of the metal–semiconductor junction, ${A}^{*}$ is the is the effective Richardson constant of the majority charge carriers, ${C}_{2}$ characterizes the barrier lowering due to the voltage drop across the interfacial layer, $\uptheta$ is the transmission coefficient across the interfacial layer, ${V}_{R}$ and ${V}_{D}$ are the thermal and diffusion velocity, respectively.

This procedure uses the $H$ function defined by:

2 $$H\left(V,T\right)=\frac{{V}_{TH}}{J}\frac{\partial J}{\partial V},$$

which is calculated from the measured I–V data.

The *H* function, applied on the *I–V* curves modelled with the ITD model theory$,$ can be written as:

3 $$H\left(V,T\right)=\frac{{V}_{TH}}{{V}_{D}}\frac{\partial {V}_{D}}{\partial V}\frac{1}{1+\frac{{V}_{D}}{\theta {V}_{R}}}+{C}_{2}.$$

If thermionic mechanism (TE) dominates the current, i.e. ${\theta V}_{R}\ll {V}_{D}$, the $H$ function can be approximated as follows:

4 $$H\left(V,T\right)={C}_{2}.$$

The presence of a plateau zone on the $H$ function allows a simple and well-defined identification of the TE voltage range. Therefore, the behaviour of the H–V curves can be used to estimate the ${C}_{2}$ parameter and identify the I–V zone dominated by the TE mechanism. The knowledge of both ${C}_{2}$ and the current values in the TE regime is key in the estimation of further characteristic parameters of the electrical contacts. The slope and the intercept of the linear Arrhenius plots of $ln(J/{T}^{2})-{C}_{2}V/{V}_{TH}$ versus $q/KT$ give the estimation of the barrier height ${\phi }_{{B}_{0}}$ and the product ${A}^{*}q$. Hence, the $H$ function allows us to determine the TE voltage range and to estimate the contact characteristic parameters by taking into account the barrier lowering (through the parameter ${C}_{2}$).

### Structural characterization

Two additional samples equipped with platinum contacts (both PPs type) were realised and dedicated to a in-depth structural and morphological characterization. Cross-sectional Transmission Electron Microscopy (TEM) and Energy Dispersive X-ray Spectroscopy (EDX) were performed to investigate the structural and chemical properties of the Pt layer and of the CZT-metal junction. EDX maps were obtained in Scanning-TEM (STEM) mode using high-angle annular dark-field (HAADF) detector. TEM measurements were performed on a JEOL 2200FS microscope operating at 200 kV, equipped with an Energy Dispersive X-ray spectrometer (EDX), an in-column energy Omega filter, and a High-Angle Annular Dark-Field (HAADF) detector.

**Figure 2:**
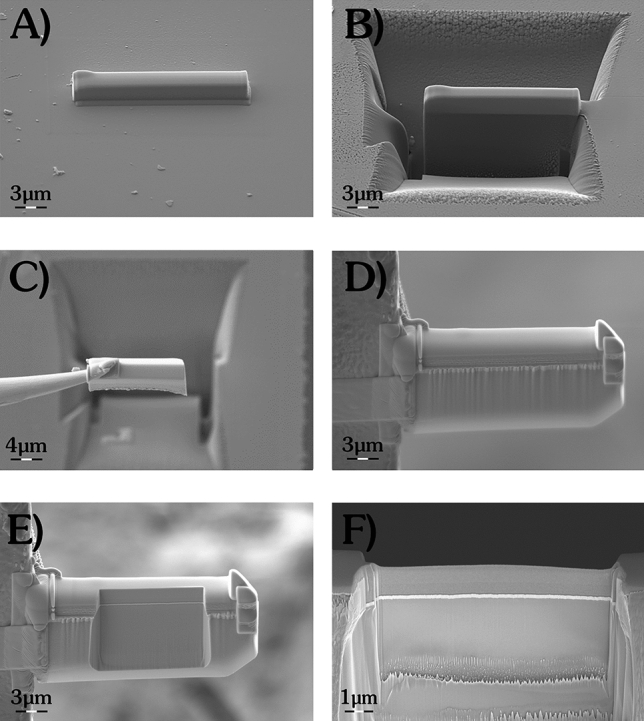
SEM images of different steps during FIB preparation of the CZT-lamella for TEM analysis: (**A**) selection of the site of interest for the lamella preparation and FIB-deposition of a protective layer of Pt; (**B**) FIB milling of the regions around the lamella; (**C**) micromanipulator assisted lift-out of the lamella; (**D**) positioning of the lamella on the TEM grid; (**E**) initial and (**F**) final phase of FIB thinning of the lamella to the electron transparency.

Since TEM works in transmission mode, samples had to be thinned down to few tens of nanometers. A technique to achieve this thickness is the preparation of a “lamella” produced by means of Focused Ion Beam (FIB) lift-out technique. Figure 2 shows the Scanning Electron Microscopy (SEM) imaging of subsequent steps of the FIB preparation of a TEM-lamella on the CZT sample. FIB lamella preparation and SEM imaging were performed with a Zeiss Auriga Compact Crossbeam system equipped with a Gemini electron column, a Canion ionic column with Ga source, a Gas Injection System with Pt-precursor reservoir and a Kleindiek micromanipulator.

### X-ray and gamma ray measurements

The spectroscopic performances of the detectors were investigated at DiFC of University of Palermo (Italy). The detectors were irradiated through the cathode electrode with uncollimated radiation sources (main gamma lines: ^241^Am, 59.5 keV and 26.3 keV; ^57^Co, 122.1 keV and 136.5 keV; ^109^Cd: 22.1 and 24.9 keV). We used two different ^241^Am sources, one also emitting the Np L X-ray lines (13–21 keV), and the other one with these lines shielded by the source capsule. All measurements were performed at room temperature (T = 20 °C). To measure the energy spectra, the detectors were connected to custom charge sensitive preamplifiers (CSPs), characterized by an equivalent noise charge (ENC) of about 100 electrons (equivalent to about 1 keV FWHM for CZT detectors) and equipped with RC feedback module with exponential decay and time constant of 100 µs. The CSP output pulses were processed with a commercial shaping amplifier (672 Spectroscopy Amplifier, Ortec/AMETEK, USA) and a multichannel analyzer (MCA 8000D, Amptek, USA). A shaping time constant of 1 µs was used. The energy resolution was estimated by using a dedicated best fitting function which also considers the asymmetry of the energy peaks^24^.

## Results

### Electrical properties

**Figure 3:**
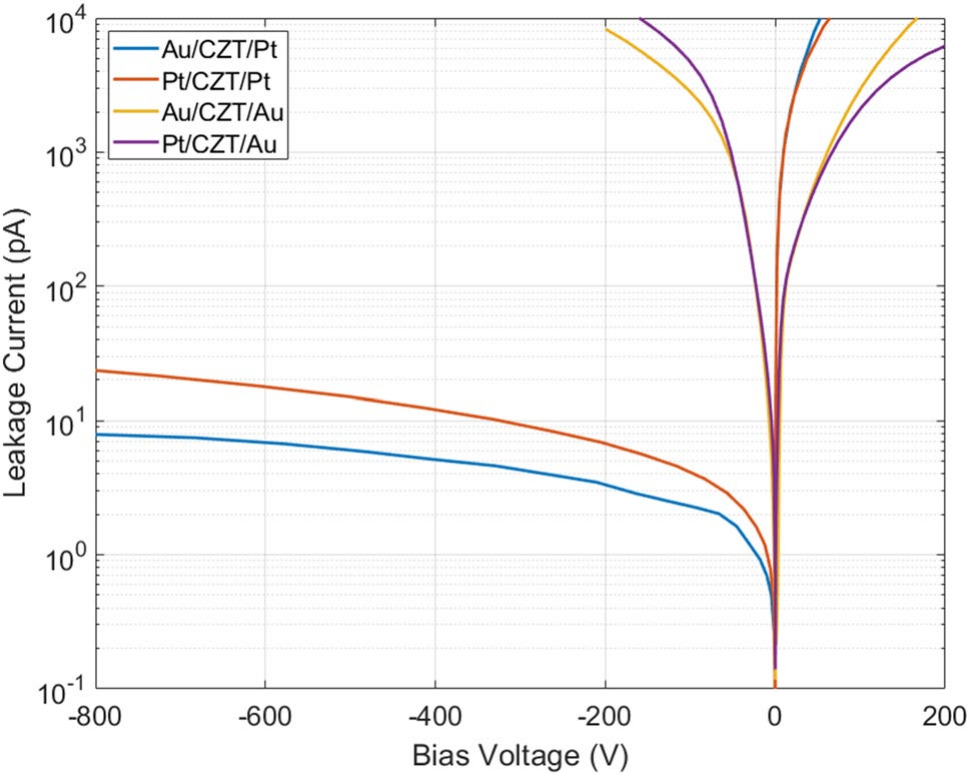
Current–voltage curves of HF-CZT samples AA, AP, PA and PP.

Room-temperature I-V curves of the four samples are shown in Fig. 3. The plot shows the behaviour of the sensors in dark condition. The I–V curves were obtained by measuring the leakage current of one of the two isolated single pixels for each sample, while guard-pad was grounded.

Voltage range for AA (Au/CZT/Au) and PA (Pt/CZT/Au) detectors was limited to − 200 ÷ 200 V due to extremely high leakage currents (> 10^4^ nA). The two samples with platinum on Cd-face (AP and PP) instead present low dark leakage current even at high negative voltage bias. The strong asymmetry between the positive and negative branches of these two samples is noteworthy. The low dark leakage current in reverse bias condition, corresponding to a current density of about 40 pA/mm^2^ at a polarization of 500 V/mm, makes Au/CZT/Pt (AP) and Pt/CZT/Pt (PP) the most promising metal electrode combination.

From the analysis of the I–V curves shown in Fig. 3 it is possible to state the two following conclusions. First, the contact that determines the reverse leakage current of the detector is the one on the Cd-face. Indeed, this current is very high when gold is chosen as metal for the Cd-face, while it is very low when this contact is made of platinum. Second, the metal contact chosen for the Te-face does not affect the I-V shape.

Considering that (a) in reverse bias condition, the injected carriers on Cd-face are holes, while injected carriers on Te-face are electrons, and (b) the I–V curves, for fixed metal contact on the Cd-face, do not change significantly by varying the metal on the Te-face, we make the hypothesis that the major contribution to dark leakage current for these four samples is due to holes. Since sample PA and AA (gold on Cd-face) present high reverse leakage currents, we deduce that the gold contact does not block holes injection.

**Figure 4:**
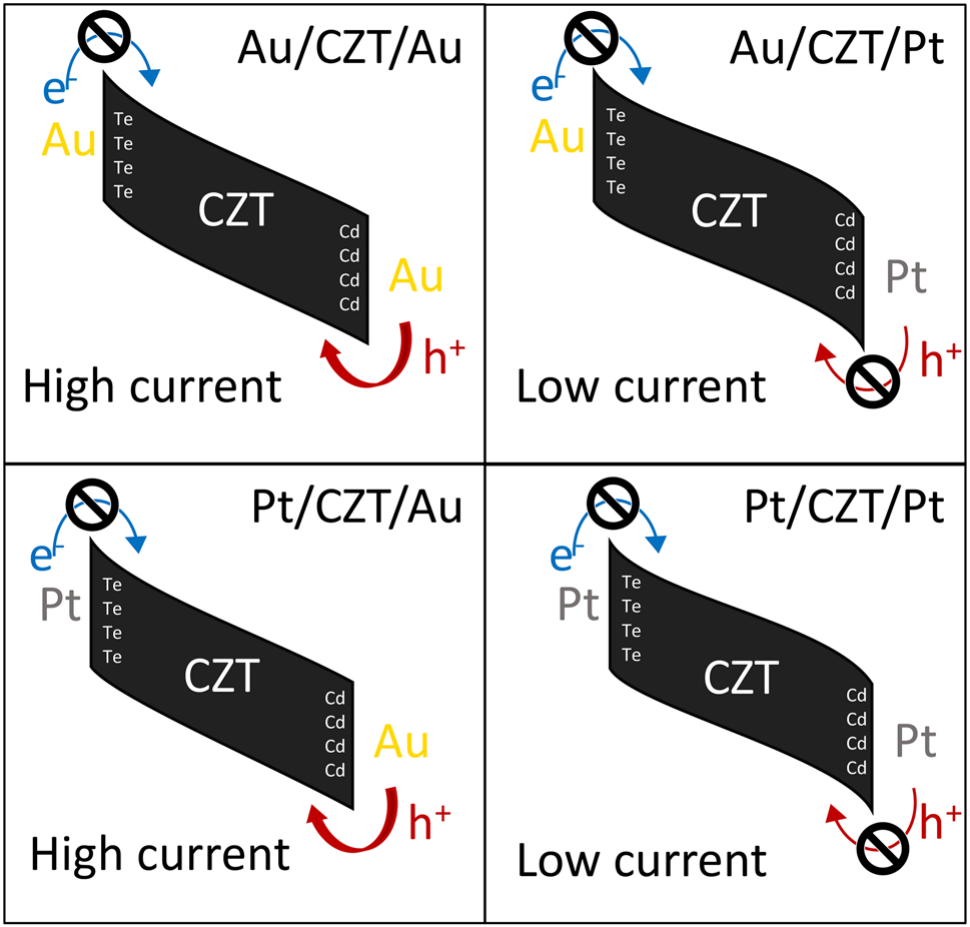
Band diagram sketch of the four different detectors in reverse bias condition. The “denied access” symbol means that the contact blocks carrier injection.

Figure 4 is a tentative schematic representation of the band diagram of each detector in reverse bias, as it can be deduced by previous statements.

By looking at the first row (Fig. 4a,b) or at the second row (Fig. 4c,d), it can be inferred that Pt on the anode blocks hole injection, while Au does not. However, in the situations Fig. 4b,d, both contacts are blocking, so it is unknown whether electron or hole current prevails. By comparing Fig. 4b,d, it can be noticed that changing the cathode electrode, responsible for electrons injection, the result does not change. Thus, we deduce that the major contribution to the dark reverse leakage current for this type of HF-CZT is given by holes.

The best band diagram, i.e., the one with the lowest possible dark noise, prevents holes and electrons to be injected from outside when the sensor is biased in the absence of radiation, but, at the same time, enables holes and electrons to exit when they are generated inside the CZT crystal by radiation absorption, to avoid internal self-polarisation of the crystal.

Among the four depicted in Fig. 3, the two on the right column (AP and PP) correspond to this preferable situation.

From room-temperature I–V curves analysis, the following conclusions can be drawn when the detector is reverse biased:

- Gold on Cd-face does not block the injection of holes, which likely constitute the major contribution to the total dark leakage current.
- Platinum on Cd-face blocks the injection of holes to a greater extent, even though holes current remains the major contribution to the total current.
- Both gold and platinum on Te-face block the injection of electrons.

To confirm the hypothesis that hole current is the main contribution to the total dark leakage current, I–V curves at different temperature on AP and PP samples were performed. This allowed us to better understand the contact nature and extract contact parameters such as barrier height and transmission coefficient, by analysing them with ITD model and $H$ function. Assuming that current is driven by holes for both samples, we expect the match of the values of contact parameters of sample AP and PP.

**Figure 5:**
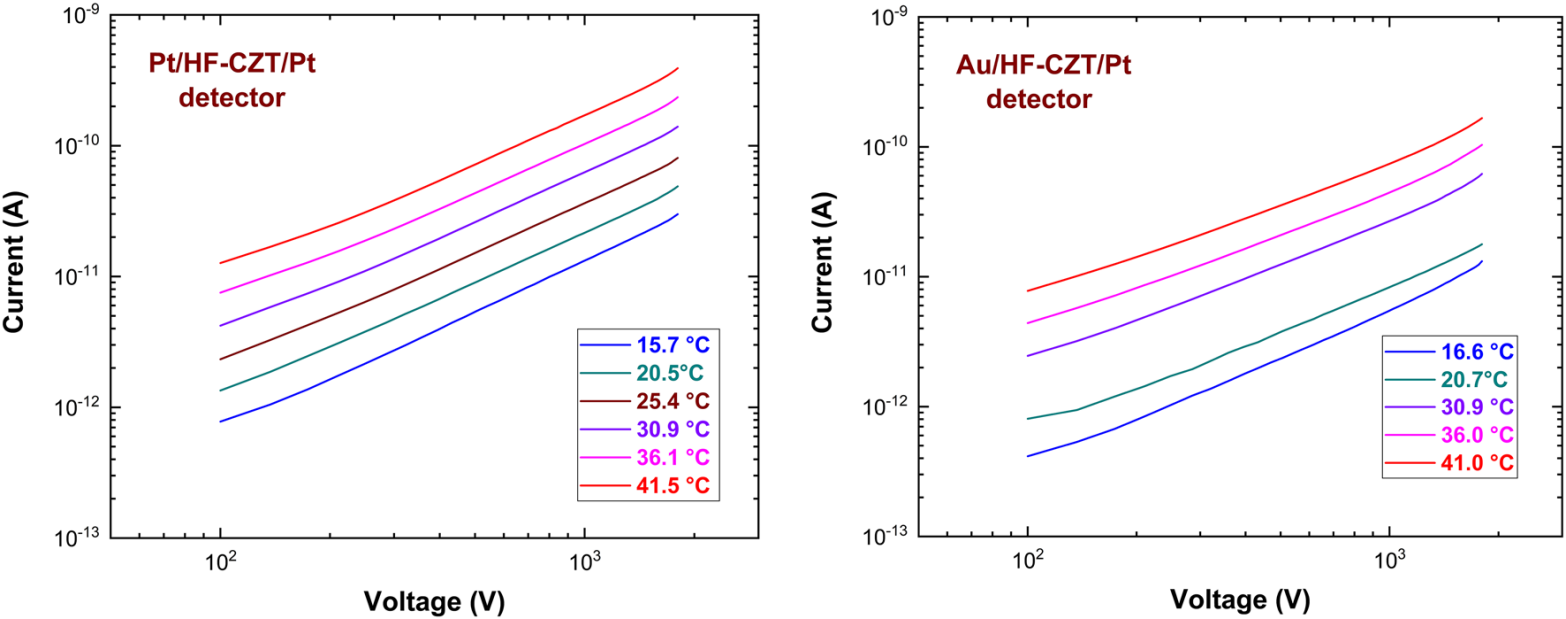
Measured I–V curves at different temperatures for a pixel of PP (left) and of AP (right) detector.

**Figure 6:**
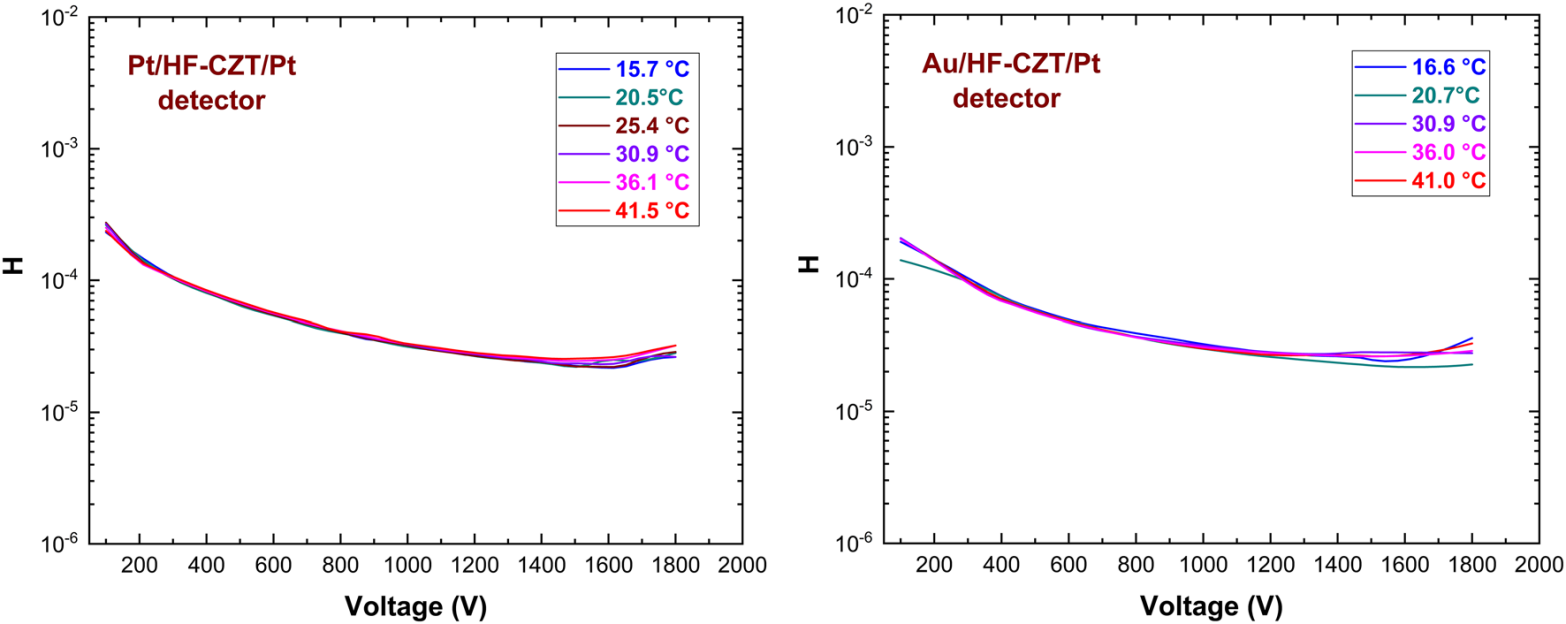
Experimental H–V curves at different temperatures of PP (left) and AP (right) detectors.

**Figure 7:**
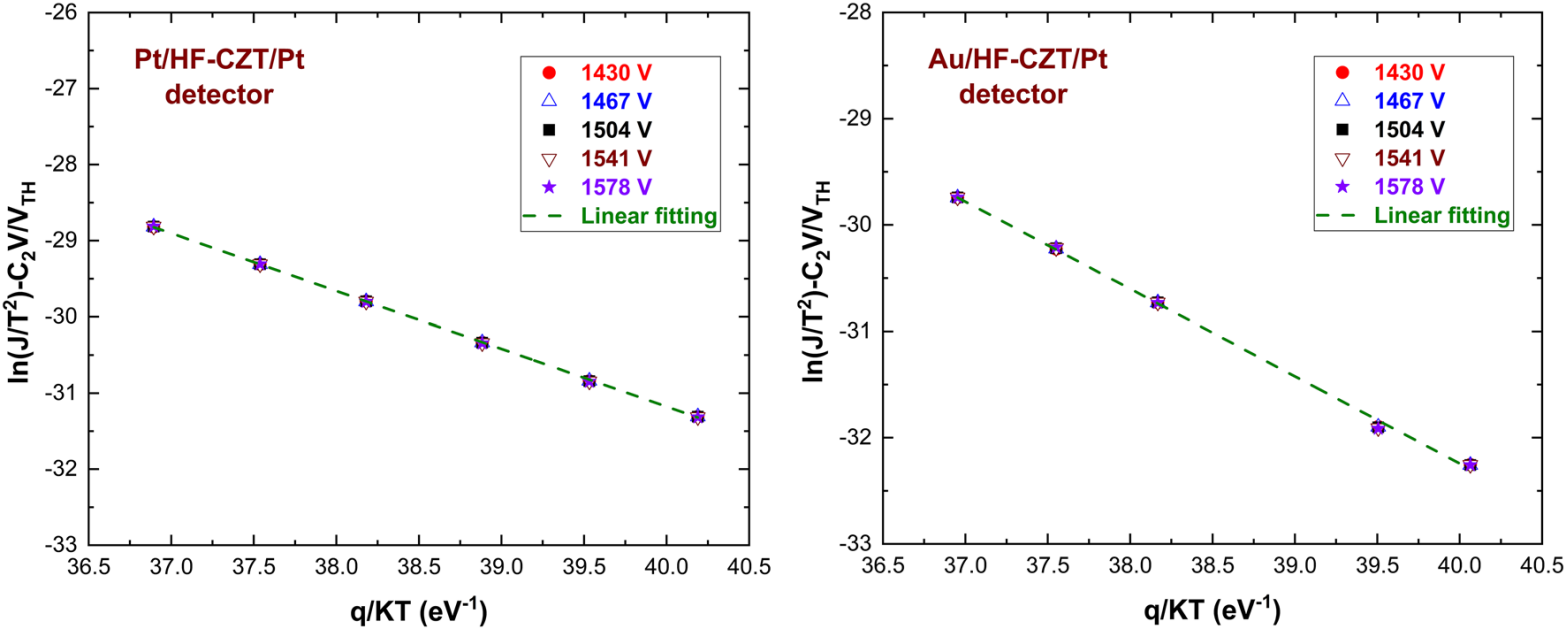
Experimental Arrhenius plots of ${\text{ln}}(J/{T}^{2})-{C}_{2}V/{V}_{TH}$ versus $q/KT$, at different bias voltages.

ITD model parameters were calculated for three different pixels on each AP and PP sample to enhance the accuracy of the results. Only three pixels among six of each detector have been measured, having the same area, so that current values can be directly comparable. Figure 5 shows the I–V curves of the investigated detectors at different temperatures, measured in reverse biasing from − 100 to − 1800 V. The I–V curves are here shown for one pixel only of each detector. The experimental $H$ function values versus the bias voltage are shown in Fig. 6. Generally, the H-V curves follow the expected behaviour from the ITD model. These curves are quite independent of the temperature, showing decreasing values with voltage raising and reaching a plateau above 1400 V. Figure 7 shows the Arrhenius plots of $ln(J/{T}^{2})-{C}_{2}V/{V}_{TH}$ versus $q/KT$, obtained at different bias voltages, properly selected in the plateau zone of the related H–V curves. The linear behaviour is visible, and quite good independence from the voltage is generally observed. The barrier height ${\phi }_{{B}_{0}}$ and the transmission coefficient ${\theta }_{p}$ values were calculated from the Arrhenius plots at all voltages located in the plateau zone.

Table 1 summarises the estimated ${C}_{2}$ and barrier height ${\phi }_{{B}_{0}}$ values, obtained from H–V curves. Results calculated from I–V of three different pixels for each sample were reported.

**Table 1:** Parameters obtained by curve fitting of IV measured for different pixels of the two samples.

Sample and pixel ID	Barrier height ${\phi }_{{B}_{0}}$ [meV]	${C}_{2}$ parameter [10^−6^]	Transmission coefficient ${\theta }_{p}$ [10^−2^]
PP—pixel 1	770 ± 10	23 ± 2	17 ± 1
PP—pixel 2	760 ± 10	25 ± 1	12.1 ± 0.5
PP—pixel 3	760 ± 10	25 ± 1	12.2 ± 0.7
AP—pixel 1	770 ± 20	19 ± 5	24 ± 2
AP—pixel 2	790 ± 40	23 ± 3	42 ± 3
AP—pixel 3	810 ± 40	26 ± 3	52 ± 3

The hole barrier height of platinum contact calculated from all measured I–Vs is about 770 meV. This is the first time that the barrier height of platinum contacts on CZT has been calculated for holes. The estimate value of the ${C}_{2}$ parameter is almost the same for all investigated samples and is of the same order of magnitude as that estimated for other Pt/CZT detectors^11^. ${\theta }_{p}$ value depends on interfacial layer characteristics The fact that ${\theta }_{p}$ values calculated from pixels of the same detectors are marginally different is probably due to a light spatial inhomogeneity of the interfacial layer.

Data obtained from different pixels and detectors are similar, meaning that the hypothesis made in previous paragraphs is verified. Dark leakage current in these samples is dominated by the hole’s contribution and platinum deposited on Cd-face is necessary to limit its injection.

The ITD model predicts that, in the limit of no oxide layer, the coefficient ${C}_{2}$ would be zero and the parameter ${\theta }_{p}$ would be equal to one. The values reported in Table 1 are far from these values, suggesting that an oxide interfacial layer is present. To verify and investigate the presence of this layer, the sputtered Pt contact on CZT was investigated using transmission electron microscopy (TEM).

### TEM analyses

The structural and chemical properties of Pt contacts on CZT, both on Cd-face and Te-face, have been studied at the nanoscale level by Transmission Electron Microscopy. A lamella of the Pt contact was prepared using focused ion beam (FIB) lift-out technique and then analysed by TEM. Since the results were similar, only the Cd-face data are shown (data concerning Pt on Te-face are presented in the Supplementary Info).

**Figure 8:**
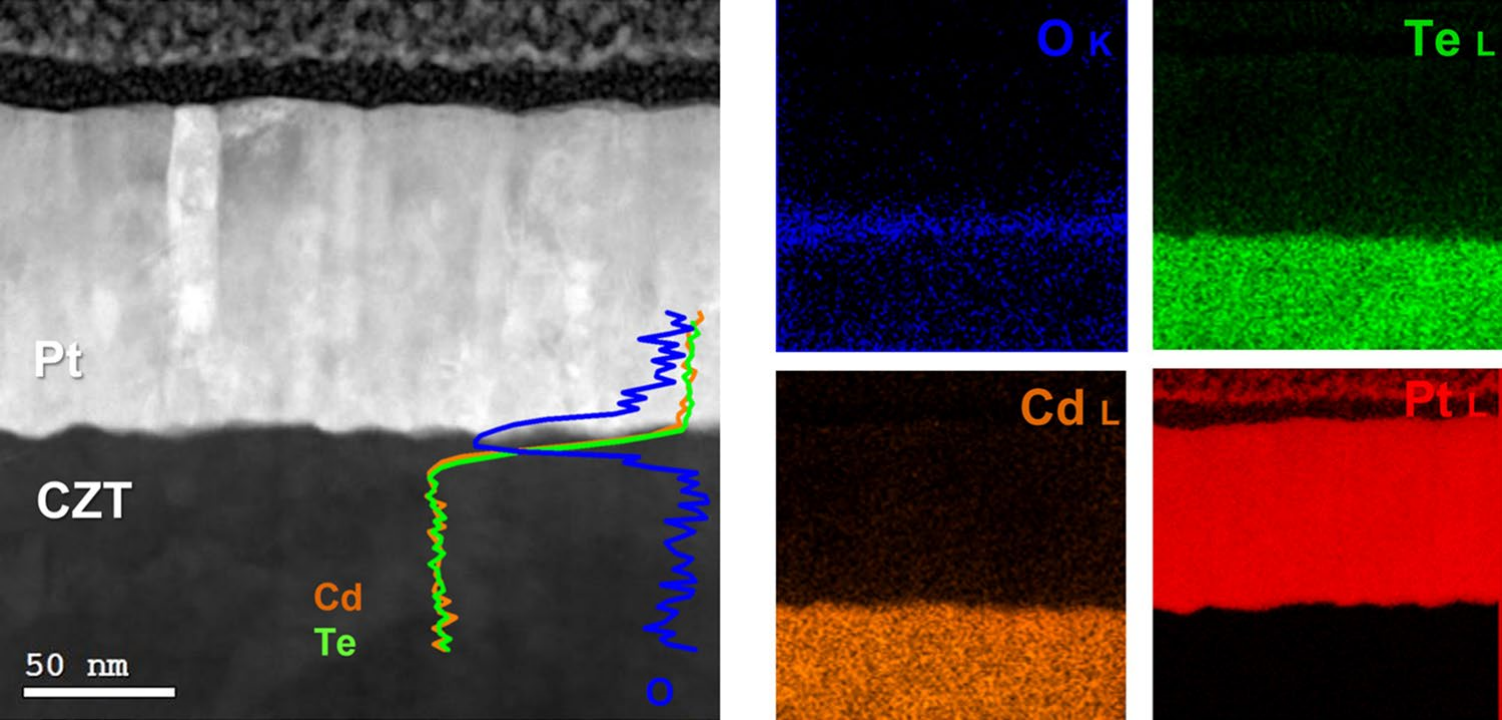
(Left) Cross section HAADF-STEM image of the Pt/CZT contact at the anode, with the overlaid EDX line-scan profiles across the interface. (Right) the corresponding Cd, Te, Pt and O EDX maps.

Figure 8 shows a representative cross section HAADF image of the Pt/CZT contact at the Cd-face (anode) and the corresponding EDX maps of Cd, Te, Pt and O atoms. An oxygen-rich interlayer at the Pt/CZT interface can be clearly observed by overlapping the STEM-EDX line profile to the HAADF image, showing the in-depth distribution of the elements. Cd and Te were found in the 1:1 ratio throughout the interlayer, as determined by quantitative EDX.

**Figure 9:**
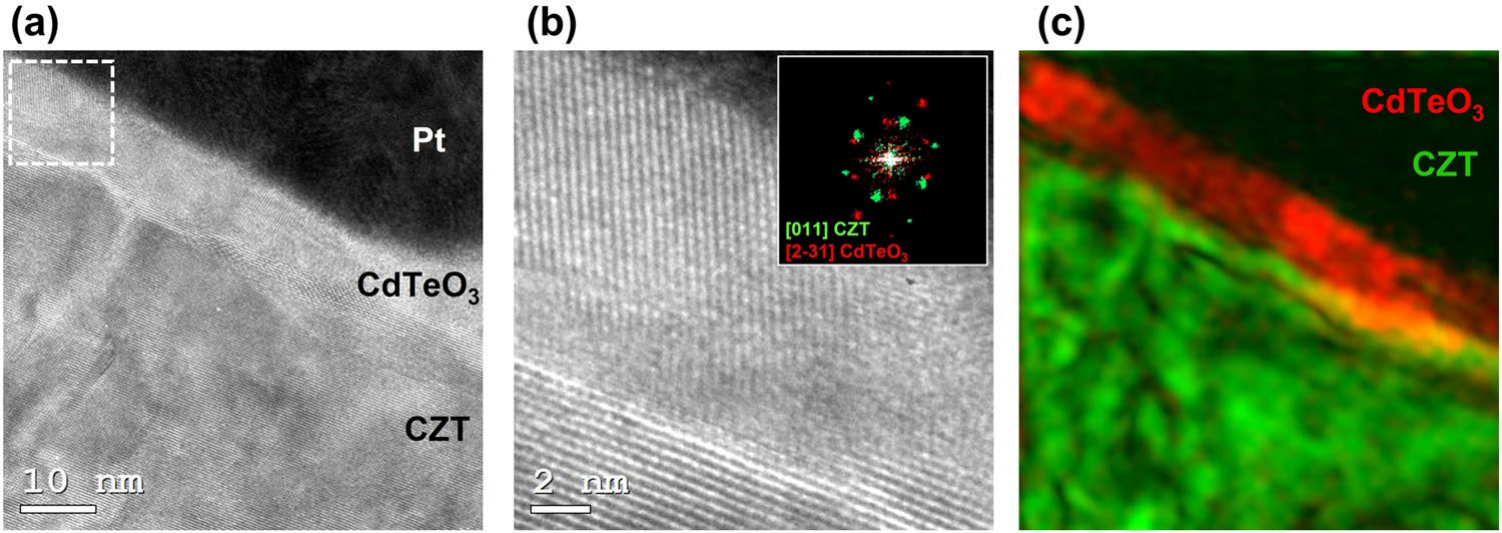
(**a**) Cross section HRTEM image of the Pt/CZT contact at the anode. (**b**) Enlarged image of the dotted square in (**a**) with the corresponding FFT pattern in the inset, showing the spots of the CZT in the [011] zone axis (green) and the spots of the orthorhombic CdTeO_3_ in the <231> zone axis (red). (**c**) Color map displaying the inverse FFT generated by selecting the reflections relative to the CZT and the oxide layer.

Direct evidence of the oxide layer was provided by HRTEM analysis. Figure 9a shows the presence of a layer of about 10 nm at the Pt/CZT interface, whose crystalline structure differs from that of the underlying CZT, as clearly visible by enlarging the area marked by the dotted line (Fig. 9b).

The nature of the thin layer was identified by Fast Fourier Transform (FFT) analysis (inset of Fig. 9b). Among all Cd and Te oxides, the FFT pattern of the layer (red spots) was uniquely attributed to the CdTeO_3_ oxide. This finding is in agreement with the literature^25,26^ and with the EDX quantitative analysis. The distribution of the oxide and CZT is shown in the colour map of Fig. 9c displaying the inverse FFT generated by selecting the reflections relative to the two phases.

The presence of the CdTeO_3_ oxide was also detected at the Pt/CZT contact at the cathode (Te-face), on which the same TEM analysis was carried out (see Fig. SI_2 in the Supplementary Information).

### Spectroscopic performance

The AP and PP detectors were successively bonded on a dedicated PCB to test their spectroscopic performance.

**Figure 10:**
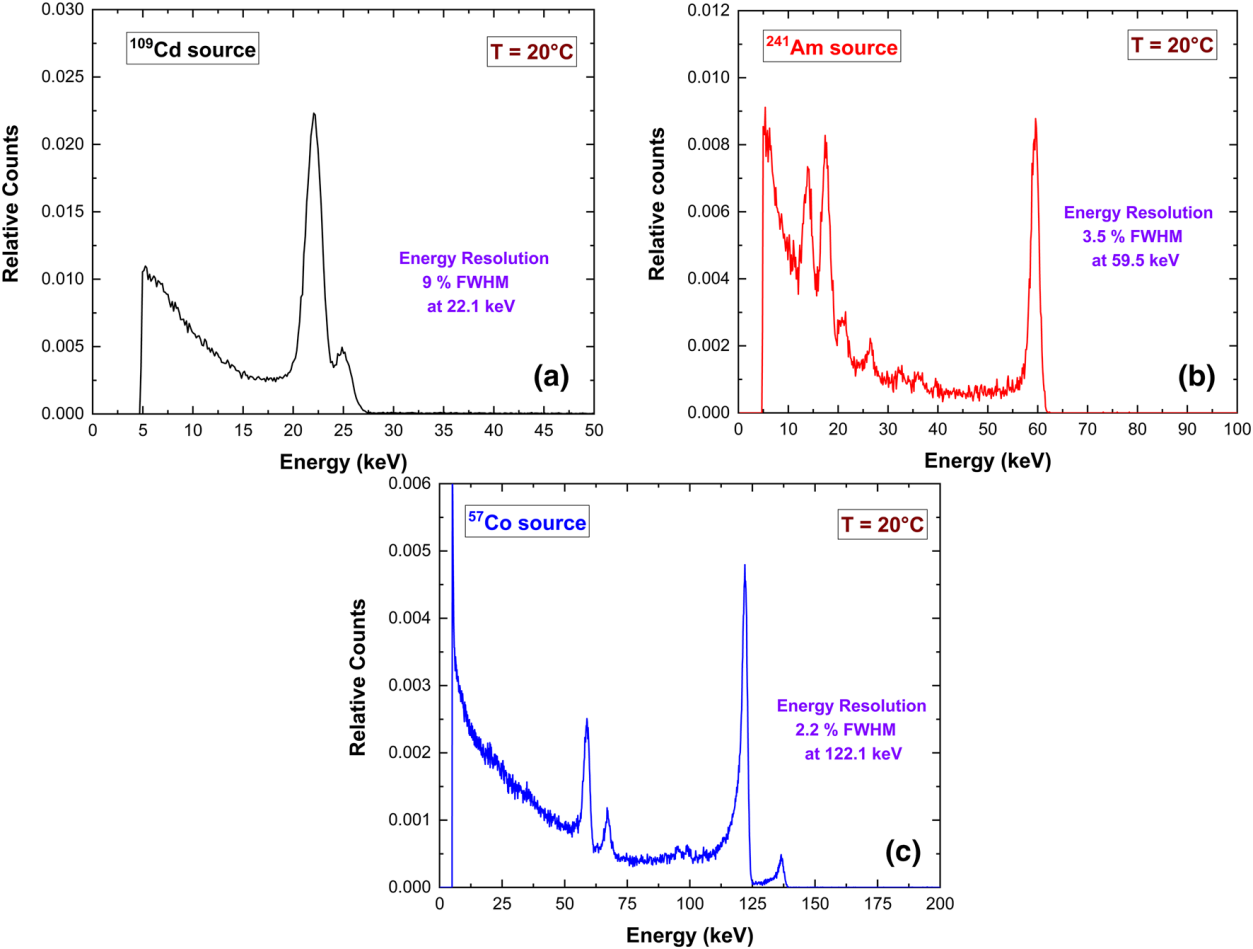
(**a**) ^109^Cd, (**b**) ^241^Am, (**c**) ^57^Co energy spectra of a tested pixel of the AP detector.

**Figure 11:**
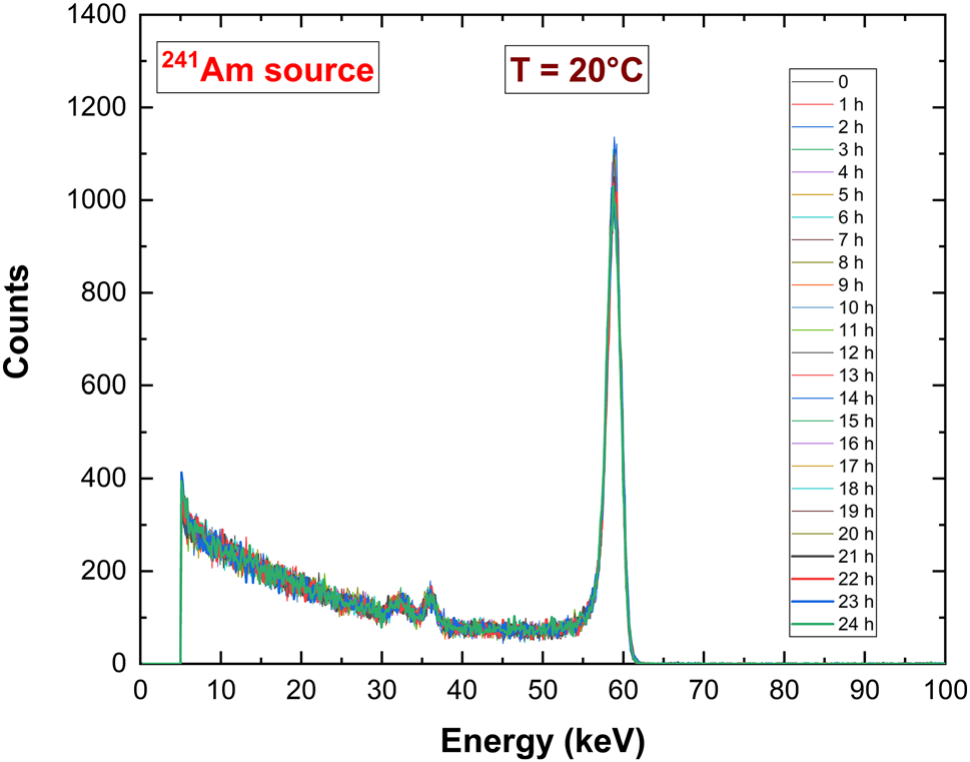
Time stability of ^241^Am energy spectra.

The spectroscopic performances of the detectors were investigated at room temperature (T = 20 °C) by using uncollimated radiation sources (^109^Cd, ^241^Am, ^57^Co). Since the spectroscopic performances of the two detectors were similar, only the energy spectra obtained with AP sample are shown in Fig. 10 (bias voltage of − 700 V). The spectra are characterized by low tailing, due to the good transport properties of holes. Time stability of the detector was verified, spectra achieved from 24 h of measurements were collected and reported in Fig. 11.

AP and PP detectors were also tested under high flux condition (up to 1 × 10^12^ photons/mm^2^/s), a deep characterization under high photon flux was carried out and results are reported in the work of Baussens et al.^5^. Both samples showed excellent linearity response and time stability under high flux, making platinum contacts very appealing for detector fabrication using HF-CZT.

## Discussion

The behaviour of gold electroless and platinum sputtered contacts deposited on HF-CZT was the main object of this study.

Experimentally, we found that dark reverse leakage current is mainly due to holes, and that a platinum contact on the Cd-face (used as anode) is needed to limit hole injection during detector operation. Therefore, platinum contacts on high-flux CZT were extensively studied. I–V curves were measured at several temperatures and, by using the $H$ function, the characteristic parameters of the contact were obtained. The hole barrier height is about 770 meV while ${\theta }_{p}$ and ${C}_{2}$ values indicate the presence of an oxide layer between CZT and platinum. Thanks to TEM analysis an interfacial layer of crystalline CdTeO_3_ was found. The oxide layer is approximately 10 nm thick, and shows orthorhombic structure with orientation along the <231> zone axis. This orientation minimises the lattice mismatch between oxide layer and CZT. The optimized contacts allow to polarize the detectors at high voltages keeping low values of the leakage current (300 pA/mm^2^ @ 1200 V/mm). The possibility to operate with high voltages is crucial under high flux, where the collection of photogenerated carriers must be as efficient as possible. The realised detectors also show a great electrical and spectroscopic time stability and high spectroscopic performances. In a parallel work^5^, contact quality was also verified under high photon flux (up to 1 × 10^12^ photons/mm^2^/s) obtaining a very good linearity response.

As reported in our previous work^16^, the electroless deposition of platinum on CZT does not lead to the formation of the oxide layer. This could be due to the chemical condition created during the deposition that brings to the formation of PtTe_2_ alloy instead of CdTeO_3_ oxide. Since tests with electroless platinum layer on high-flux CZT have been unsuccessful, even though the deposited metal is the same, we suppose that the combination of interfacial CdTeO_3_ and platinum ensures the optimal band structure alignment at the interface, which blocks hole injection and facilitates the extraction of photogenerated carriers, maximizing in such way the signal to noise ratio.

### Supplementary Information

Supplementary Figures.

## Data Availability

All data necessary to replicate the experiments are included in the paper. Datasets generated and acquired during the current study are available from the corresponding author on a reasonable request.

## References

[1] Iniewski K (2016). CZT sensors for computed tomography: From crystal growth to image quality. J. Instrum..

[2] Prokesch M, Soldner SA, Sundaram AG (2018). CdZnTe detectors for gamma spectroscopy and X-ray photon counting at 250 × 106 photons/(mm^2^ s). J. Appl. Phys..

[3] Veale MC (2019). Cadmium zinc telluride pixel detectors for high-intensity X-ray imaging at free electron lasers. J. Phys. Appl. Phys..

[4] Veale MC (2020). Characterization of the uniformity of high-flux CdZnTe material. Sensors.

[5] Baussens O (2022). Characterization of high-flux CdZnTe with optimized electrodes for 4th generation synchrotrons. J. Instrum..

[6] Thomas B (2017). Characterisation of Redlen high-flux CdZnTe. J. Instrum..

[7] Li Y (2021). Effects of deep-level traps on the transport properties of high-flux X-ray CdZnTe detectors. Mater. Sci. Semicond. Process..

[8] Abbene L (2018). Dual-polarity pulse processing and analysis for charge-loss correction in cadmium–zinc–telluride pixel detectors. J. Synchrotron. Radiat..

[9] Buttacavoli A (2022). Incomplete charge collection at inter-pixel gap in low- and high-flux cadmium zinc telluride pixel detectors. Sensors.

[10] Ünal M, Turan R, Abbene L, Iniewski K (2023). A path to produce high-performance CdZnTe crystals for radiation detection applications: Crystal growth by THM, surface preparation, and electrode deposition. High-Z Materials for X-ray Detection: Material Properties and Characterization Techniques.

[11] Bolotnikov AE (2002). Properties of Pt Schottky type contacts on high-resistivity CdZnTe detectors. Nucl. Instrum. Methods Phys. Res. Sect. Accel. Spectrom. Detect. Assoc. Equip..

[12] Turturici AA (2016). Electrical properties of Au/CdZnTe/Au detectors grown by the boron oxide encapsulated Vertical Bridgman technique. Nucl. Instrum. Methods Phys. Res. Sect. Accel. Spectrom. Detect. Assoc. Equip..

[13] Benassi G (2017). Strong mechanical adhesion of gold electroless contacts on CdZnTe deposited by alcoholic solutions. J. Instrum..

[14] Yu J, Xu L, Zhang B, Zha G, Jie W (2020). On the current transport mechanism in metal–semiconductor–metal structured CdZnTe radiation detectors. Nucl. Instrum. Methods Phys. Res. Sect. Accel. Spectrom. Detect. Assoc. Equip..

[15] Yu J, Xu L, Li Y, Zha G, Jie W (2021). Bias-induced relaxation phenomena in current temporal behaviors of CdZnTe radiation detectors. Nucl. Instrum. Methods Phys. Res. Sect. Accel. Spectrom. Detect. Assoc. Equip..

[16] Bettelli M (2020). Improved electroless platinum contacts on CdZnTe X- and γ-rays detectors. Sci. Rep..

[17] Principato F, Bettelli M, Zappettini A, Abbene L (2023). A novel extraction procedure of contact characteristic parameters from current–voltage curves in CdZnTe and CdTe detectors. Sensors.

[18] Abbene L (2018). Digital fast pulse shape and height analysis on cadmium–zinc–telluride arrays for high-flux energy-resolved X-ray imaging. J. Synchrotron. Radiat..

[19] Buttacavoli A (2020). Room-temperature performance of 3 mm-thick cadmium–zinc–telluride pixel detectors with sub-millimetre pixelization. J. Synchrotron. Radiat..

[20] Tsigaridas S (2021). Fabrication of small-pixel CdZnTe sensors and characterization with X-rays. Sensors.

[21] Chen, H. *et al.* CZT device with improved sensitivity for medical imaging and homeland security applications. In *Hard X-Ray, Gamma-Ray, and Neutron Detector Physics XI* Vol. 7449, 15–31 (SPIE, 2009).

[22] Wu C (1982). Interfacial layer-thermionic-diffusion theory for the Schottky barrier diode. J. Appl. Phys..

[23] Wu C (2008). Interfacial layer theory of the Schottky barrier diodes. J. Appl. Phys..

[24] Del Sordo S (2004). Spectroscopic performances of 16×16 pixel CZT imaging hard-X-ray detectors. Il Nuovo Cimento B.

[25] Zha G, Jie W, Tan T, Zhang W, Xu F (2007). The interface reaction and Schottky barrier between metals and CdZnTe. J. Phys. Chem. C.

[26] Guillén-Cervantes A (2020). Structural and optical properties of CdTe + CdTeO3 nanocomposite films with broad blueish photoluminescence. J. Mater. Sci. Mater. Electron..

