# Supplementary material for: High performance platinum contacts on high-flux CdZnTe detectors
Source: Sci Rep. 2023 Oct 20;13:17963. doi: 10.1038/s41598-023-45331-9 (PMC10589347; doi:10.1038/s41598-023-45331-9)
Supplement: Supplementary file 1 — Supplementary Figures. [file 41598_2023_45331_MOESM1_ESM.pdf]

Supplementary Info

In this section are collected the following images:

- SI_1: AFM image of Pt contact. The scanned area was 1000 x 1000 nm^2, and the measured surface roughness is 5.02 nm and 4.02 nm respectively for Rq and Ra.
- SI_2: Cross section HRTEM image of the Pt/CZT contact at the cathode (Te-face) with the FFT patterns taken from the oxide layer and CZT. The FFT of the oxide layer was attributed to the <231> zone axis of orthorhombic $CdTeO_3$.

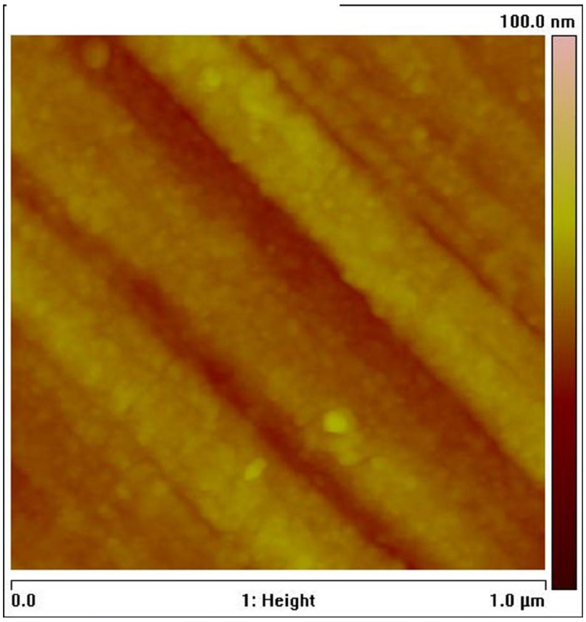

Figure SI_1. AFM image of Pt contact. The scanned area was 1000 x 1000 nm2, and the measured surface roughness is 5.02 nm and 4.02 nm respectively for Rq and Ra.

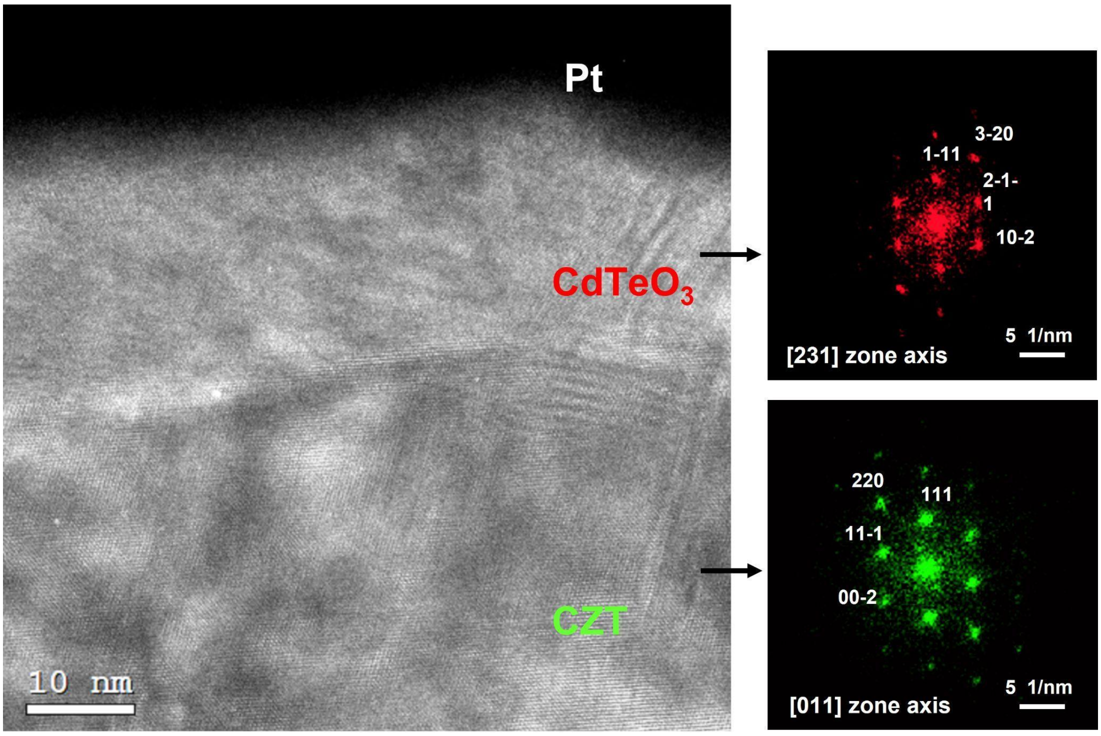

Figure SI_2. (a) Cross section HRTEM image of the Pt/CZT contact at the cathode (Te-face) with the FFT patterns taken from the oxide layer and CZT. The FFT of the oxide layer was attributed to the <231> zone axis of orthorhombic $CdTeO_3$.
